# The difficult medical emergency call: A register-based study of predictors and outcomes

**DOI:** 10.1186/s13049-017-0366-0

**Published:** 2017-03-01

**Authors:** Thea Palsgaard Møller, Thora Majlund Kjærulff, Søren Viereck, Doris Østergaard, Fredrik Folke, Annette Kjær Ersbøll, Freddy K. Lippert

**Affiliations:** 1 Emergency Medical Services Copenhagen, University of Copenhagen, Telegrafvej 5, 2750 Ballerup, Denmark; 2 National Institute of Public Health, University of Southern Denmark, Øster Farimagsgade 5A, 1353 København K, Denmark; 3 Copenhagen Academy for Medical Education and Simulation, University of Copenhagen, Herlev Ringvej 75, 2730 Herlev, Denmark

**Keywords:** Emergency call, Emergency medical dispatching, Emergency medical services, Pre-hospital emergency care, Triage

## Abstract

**Background:**

Pre-hospital emergency care requires proper categorization of emergency calls and assessment of emergency priority levels by the medical dispatchers. We investigated predictors for emergency call categorization as “unclear problem” in contrast to “symptom-specific” categories and the effect of categorization on mortality.

**Methods:**

Register-based study in a 2-year period based on emergency call data from the emergency medical dispatch center in Copenhagen combined with nationwide register data. Logistic regression analysis (N = 78,040 individuals) was used for identification of predictors of emergency call categorization as “unclear problem”. Poisson regression analysis (*N* = 97,293 calls) was used for examining the effect of categorization as “unclear problem” on mortality.

**Results:**

“Unclear problem” was the registered category in 18% of calls. Significant predictors for “unclear problem” categorization were: age (odds ratio (OR) 1.34 for age group 76+ versus 18–30 years), ethnicity (OR 1.27 for non-Danish vs. Danish), day of week (OR 0.92 for weekend vs. weekday), and time of day (OR 0.79 for night vs. day). Emergency call categorization had no effect on mortality for emergency priority level A calls, incidence rate ratio (IRR) 0.99 (95% confidence interval (CI) 0.90–1.09). For emergency priority level B calls, an association was observed, IRR 1.26 (95% CI 1.18–1.36).

**Discussions:**

The results shed light on the complexity of emergency call handling, but also implicate a need for further improvement. Educational interventions at the dispatch centers may improve the call handling, but also the underlying supportive tools are modifiable. The higher mortality rate for patients with emergency priority level B calls with “unclear problem categorization” could imply lowering the threshold for dispatching a high level ambulance response when the call is considered unclear. On the other hand a “benefit of the doubt” approach could hinder the adequate response to other patients in need for an ambulance as there is an increasing demand and limited resources for ambulance services.

**Conclusions:**

Age, ethnicity, day of week and time of day were significant predictors of emergency call categorization as “unclear problem”. “Unclear problem” categorization was not associated with mortality for emergency priority level A calls, but a higher mortality was observed for emergency priority level B calls.

## Background

In the event of acute illness or injury, citizens get access to pre-hospital emergency care through contact to emergency medical dispatch centers (EMDC). Medical emergency calls are the key to pre-hospital emergency care and the importance of this link is increasingly acknowledged in the medical literature [1, 2]. One core task for medical dispatchers is to handle emergency calls and prioritize the response for the patient. This implies identifying the nature of the problem presented by the caller and risk stratification. The ability to ask the right questions is based on various solutions, often a supportive decision tool, combined with the dispatchers’ medical knowledge. If the character and severity of the problem is identified, the dispatcher can ensure the proper response: from the guidance to the caller or patient to the pre-hospital care at the scene and ultimately for preparation of acute medical teams in the hospital emergency departments.

Studies have shown that medical dispatchers’ recognition of time-critical conditions during emergency calls is important for patients’ outcome [3–5]. Recognition of cardiac arrest is difficult; however necessary to initiate dispatcher assisted cardiopulmonary resuscitation (CPR) [6]. Despite experienced medical dispatchers and use of the supportive decision tool Danish Index for Emergency Care [7], “Unclear problem” is a common categorization of emergency calls in the Copenhagen area, counting for 19% of calls [8]. Literature regarding difficult calls is sparse; however, similar figures are reported from other emergency medical services (EMS) in Scandinavia [7, 9, 10]. If the cause is not clarified, risk stratification may be less sensitive, possibly leading to provision of either a higher or a lower emergency priority level than needed, resulting in unnecessary high response or lack of an appropriate high priority response.

The underlying mechanisms for the categorization of emergency calls as “unclear problem” as opposed to “symptom-specific” categories (hereafter referred to as “call_unclear_” and “call_specific_”, respectively) are unidentified, but influencing factors may be related to the patient, caller, medical dispatcher, logistic infrastructure or emergency medical services (EMS) system. Moreover, the clinical implications of this categorization are unknown.

The aim of the study was to identify patient- and EMS-related predictors for emergency calls being categorized as call_unclear_ as opposed to call_specific_ and to investigate the effect of emergency call categorization as call_unclear_ on mortality. Additionally, we explored the primary registered diagnoses for patients referred to hospitals within 12 h following an emergency call.

## Methods

A register-based study in a 2-year study period (December 1, 2011 – November 30, 2013) based on emergency call data from EMS, Copenhagen, the Capital Region of Denmark was performed.

### Setting

In Denmark a single emergency phone number (1-1-2) leads to a primary call center for emergency police, fire, or medical requests, manned by police or fire personnel. In case of a medical issue, the call is redirected to an EMDC in one of five regional EMS. The Capital Region of Denmark covers an area of 2,549 km^2^ and has a population of 1.75 million inhabitants. At the EMDC medical dispatchers prioritize the call and provide pre-arrival instructions to the caller when appropriate. The medical dispatchers are either paramedics or registered nurses, trained to handle emergency calls and register relevant data (including dispatch codes) by use of peer training at the beginning of their employment. Dispatch processes are fully computerized with use of computer aided dispatch (Logis CAD, Logis Solutions A/S, Copenhagen, Denmark). A supportive criteria-based dispatch tool (Danish Index for Emergency Care) was implemented in Denmark in May 2011 [7] (https://www.regionh.dk/om-region-hovedstaden/Den-Praehospitale-Virksomhed/Akutberedskabets-organisation/112-AMK-Vagtcentralen/Documents/Dansk%20Indeks%20version%201.5%20-%20landsudgaven%20(enkeltsider).pdf). The system was developed in Seattle, Washington in 1990 [11] and further adapted into Scandinavian context [7, 12]. Overall the tool supports the process by translating the caller’s answers about symptoms and severity of conditions, into a recommendation for pre-hospital response and guidance. More specifically, emergency calls are categorized into 38 different main categories, including call_unclear_. The categorization is the first entrance into the system and leads to specific questions that make the dispatcher able to stratify calls into five emergency priority levels (ranging from A-E). Level A describes life threatening or potential life threatening symptoms; B comprises urgent, but not life threatening symptoms; C is non-urgent conditions requiring an ambulance; D is non-urgent conditions requiring supine patient transport; and E includes conditions requiring medical advice only. Finally, the actual dispatched response is either red response (immediate response with lights and siren), orange response (immediate response without lights and siren, yellow response (non-urgent response with available appropriate resources), green response (non-urgent), and blue response (medical advice, referral to a general practitioner etc.).

### Data collection and processing

Data were obtained from the EMDC database at the EMS, Copenhagen. We included emergency calls regarding individuals aged 18 years or above with a registered dispatch code and emergency priority level and registered valid unique personal identification number (so called civil registration system number, “CRS number”). The CRS number is assigned to all persons with residential location in Denmark at birth or at immigration [13]. For predictors of emergency call categorization as call_unclear_ and for the analysis of the primary registered diagnoses for patients brought into hospitals within 12 h, we included the first emergency call for individuals with calls received at the EMDC in the study period. In the evaluation of the effect of emergency call categorization on mortality we used all emergency calls estimated as emergency priority level A or B. The CRS number was used for linkage of emergency call data with nationwide Danish registers: the Danish Civil Registration System [13], Danish registers on personal labor market affiliation, education and income [14–16], the Danish National Patient Register [17], and the Danish Register of Causes of Death [18].

### Derived variables

From the EMDC database, we extracted the CRS number, dispatch code (which includes the main category and the emergency priority level A-E), provided response type and time stamps. Emergency call categorization was constructed as a binary variable (call_unclear_ and call_specific_) based on the registered dispatch code. The time of day were divided into daytime (7:00–14:59), evening (15:00–22:59), and nighttime (23:00–6:59). The day of week was divided into weekdays (Monday through Friday) and weekends (Saturday and Sunday).

From Danish registers, we derived the following variables: age (18–30 years, 31–65 years, 66–75 years and 76+ years); gender; civil status (single or cohabiting (married, in registered partnership or cohabiting)). Educational level was defined as either elementary school, short education (9–12 years of education), and medium/long education (over 12 year of education). Employment was divided into four categories: “employed” (including employed or receiving unemployment insurance); “unemployed” (including unemployed for over half a year, receiving social security, or receiving early retirement); “students”; and “retired” (receiving state pension or being voluntary early retired). To make income comparable and capture family size and income fluctuations over the lifespan, we calculated the equivalized household income stratified in three age groups (18–30, 31–65, >65 years) divided into income quintiles, as done earlier [19]. For measure of comorbidity we used data from the Danish National Patient Register [17] to calculate the Charlson Comorbidity Score at three levels: 0 (no comorbidity); 1 (mild comorbidity); and ≥2 (severe comorbidity) [20]. Ethnicity was divided into non-Danish (immigrants and descendants from outside Denmark) and Danish (including Greenland and Faeroe Island, which teach the Danish language in schools). Hospital diagnoses were categorized according to main chapters of the International Classification of Diseases, version 10 [21].

### Analysis

Descriptive analyses were performed by use of numbers and percentages. The incidence rate of mortality was calculated with the corresponding 95% confidence interval [22]. We used a multivariable logistic regression model to identify predictors of emergency call categorization as call_unclear_ versus call_specific_ and included all variables in the model. Poisson regression analysis was used to examine the effect of categorization of emergency calls as call_unclear_ compared to call_specific_ on mortality. Incidence rate ratios (IRR) and 95% confidence intervals were calculated. For calculation of person time at risk for death, each individual was included by the emergency call date and censored at the date of the next emergency call, death, or end of study period —whichever came first. Analyses were performed semi adjusted (adjusted for age and gender) and adjusted for confounders identified a priori (age, gender, employment status, educational level, ethnicity, comorbidity, time of day, and day of week).

To evaluate if emergency priority level modified the association between emergency call categorization and mortality, we included the interaction between emergency priority level and emergency call categorization. The hypothesis for this interaction effect was that the effect of being categorized as call_unclear_ compared to call_specific_ on risk of dying might be higher among persons assessed as emergency priority level B compared to persons assessed as emergency priority level A.

## Results

**Fig. 1:**
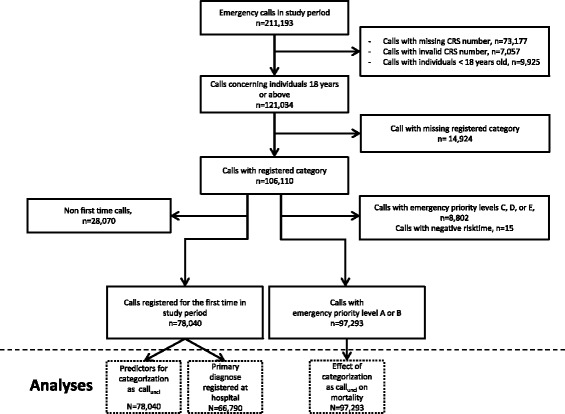
Data flowchart

Of 211,193 medical emergency calls received at the EMDC during the study period, we included 121,034 calls (Fig. 1). Among those, 78,040 persons were registered for the first time in the study period and included in the analysis of predicting factors. For the analysis of the effect of emergency call categorization as call_unclear_ on mortality, we included 97,293 individuals with emergency call assessed as emergency priority level A or B. Descriptive analysis showed that 18% of emergency calls were categorized as call_unclear_ (Table 1). Individuals with emergency calls categorized as call_unclear_ were in general older, more often of non-Danish origin, more often retired and had more comorbidities than individuals with emergency call categorized as call_specific_. Among emergency calls categorized as call_unclear_, 18.9% of the calls were estimated as emergency priority level A, compared to 47.1% of calls categorized as call_specific_. For emergency priority level A calls, a red response was provided for 96.1% of call_unclear_ and for 95.2% of call_specific_. For emergency priority level B calls, a red response was provided for 6.8% of call_unclear_ and for 6.2% of call_specific_.

**Table 1:** Frequency distribution of EMS- and patient-related characteristics for calls categorized as “unclear problem” (call_unclear_) and “symptom-specific” categories (call_specific_) given as number (*N*) and percent (%)

Variable		Call_specific_	Call_unclear_	Total
		*N* = 64,026 (82%)	*N* = 14,014 (18%)	*N* = 78,040
EMS-related				
Time of day	Daytime	28,350 (44.3)	7,023 (50.1)	35,373 (45.3)
	Evening	21,877 (34.2)	4,505 (32.2)	26,382 (33.8)
	Night	13,799 (21.6)	2,486 (17.7)	16,285 (20.9)
Time of week	Weekdays	45,459 (71.0)	10,317 (73.6)	55,776 (71.5)
	Weekend	18,567 (29.0)	3,697 (26.4)	22,264 (28.5)
Assessed emergency priority level by medical dispatcher	Level A	30,181 (47.1)	2,651 (18.9)	32,832 (42.0)
	Level B	29,604 (46.2)	9,842 (70.2)	39,446 (50.6)
	Level C	2,247 (3.5)	211 (1.5)	2,458 (3.2)
	Level D	98 (0.2)	51 (0.4)	149 (0.2)
	Level E	1,896 (3.0)	1,259 (9.0)	3,155 (4.0)
Response provided by medical dispatcher^A^	Red response	30,808 (48.1)	3,232 (23.1)	34,040 (43.6)
	Orange response	30,370 (47.4)	9,150 (65.3)	39,520 (50.6)
	Yellow response	446 (0.7)	146 (1.0)	592 (0.8)
	Blue response	2,402 (3.8)	1,486 (10.6)	3,888 (5.0)
Patient-related				
Age	18–30	10,045 (15.7)	1,585 (11.3)	11,630 (14.9)
	31–65	26,969 (42.1)	5,366 (38.3)	32,335 (41.4)
	66–75	10,519 (16.4)	2,735 (19.5)	13,254 (17.0)
	76+	16,493 (25.8)	4,328 (30.9)	20,821 (26.7)
Gender	Male	31,732 (49.5)	6,750 (48.3)	38,482 (49.3)
	Female	32,318 (50.5)	7,240 (51.8)	39,558 (50.7)
Civil status	Cohabiting	26,625 (41.6)	5,861 (41.8)	32,486 (41.6)
	Single	36,094 (56.4)	7,907 (56.4)	44,001 (56.4)
	Missing	1,307 (2.0)	246 (1.8)	1,553 (2.0)
Ethnicity	Danish origin	54,271 (84.8)	11,678 (83.3)	65,949 (84.5)
	Non-Danish origin	8,445 (13.2)	2,088 (14.9)	10,533 (13.5)
	Missing value	1,310 (2.1)	248 (1.8)	1,558 (2.0)
Educational level	Elementary school	22,910 (35.8)	4,807 (34.3)	27,717 (35.5)
	Short education	24,869 (38.8)	5,634 (40.2)	30,503 (39.1)
	Medium/long education	10,388 (16.2)	2,253 (16.1)	12,641 (16.2)
	Missing value	5,859 (9.2)	1,320 (9.4)	7,179 (9.2)
Income	1 Low	15,565 (24.3)	3,382 (24.1)	18,947 (24.3)
	2	16,250 (25.4)	3,566 (25.5)	19,816 (25.4)
	3	11,733 (18.3)	2,605 (18.6)	14,338 (18.4)
	4	9,379 (14.7)	2,033 (14.5)	11,412 (14.6)
	5 High	8,165 (12.8)	1,898 (13.5)	10,063 (12.9)
	Missing value	2,934 (4.6)	530 (3.8)	3,464 (4.4)
Employment status	Employed^B^	19,708 (30.8)	3,640 (26.0)	23,348 (29.9)
	Unemployed^C^	10,796 (16.9)	2,115 (15.1)	12,911 (16.5)
	Student	1,873 (2.9)	281 (2.0)	2,154 (2.8)
	Retired^D^	28,356 (44.3)	7,389 (52.7)	35,745 (45.8)
	“Other”	1,979 (3.1)	343 (2.5)	2,322 (3.0)
	Missing value	1,314 (2.1)	246 (1.8)	1,560 (2.0)
Comorbidity	None	32,907 (51.4)	6,800 (48.5)	39,707 (50.9)
	Mild	12,829 (20.0)	2,777 (19.8)	15,606 (20.0)
	Severe	18,290 (28.6)	4,437 (31.7)	22,727 (29.1)

^A^: Red response, acute with lights and siren; orange response, acute, no lights and siren; yellow response, transport with patient observation; blue response, medical advice, no ambulance. ^B^: employed includes employed and unemployment insurance receiver. ^C^: Unemployed includes unemployed individuals, social security recipients and individuals on early retirement. ^D^: retired includes receiving state pension or being voluntary early retired

### Predictors of unclear problems

**Fig. 2:**
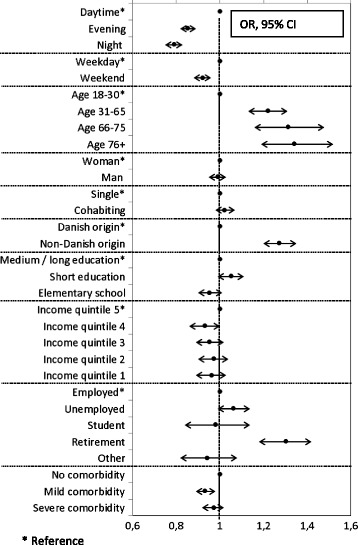
Odds ratio (OR) and 95% confidence interval (95% CI) for patient- and EMS-related characteristics predicting emergency call categorization as call_unclear_ versus call_specific_

Age, ethnicity, comorbidity, time of day, day of week, employment, and educational level were significant predictors for emergency call categorization as call_unclear_ (Fig. 2). A positive association was observed for age group 76+ versus age group 18–30 years, Odds Ratio (OR) 1.34 (95% Confidence Interval (CI) 1.19–1.51) and for non-Danish origin versus Danish origin, OR 1.27 (95% CI 1.20–1.35). A negative association was observed for nighttime versus daytime, OR 0.79 (95% CI 0.75–0.83), for weekends versus weekdays, OR 0.92 (95% CI 0.88–0.96), and for mild versus no comorbidity, OR 0.93 (95% CI 0.88–0.98). Further, educational level and employment status were significant predictors in the overall test (*p* < 0.001). Pairwise comparisons showed that persons with short education compared to all other education groups and persons retired compared to all other employment groups had significantly higher odds of being categorized as call_unclear_.

### Diagnoses for patients registered at hospital

In 66,790 (86%) cases, the patient was registered at a hospital within 12 h following an emergency call. For patients with emergency call categorization as call_unclear_ the most common diagnoses were within the main chapters “Symptoms, signs and abnormal clinical and laboratory findings, not elsewhere classified”(27%), “Factors influencing health status and contact with health services” (19%), “Injury, poisoning and certain other consequences of external causes” (12%), and “Diseases of the circulatory system” (9%). A different pattern was seen for calls categorized as call_specific_ where “Injury, poisoning and certain other consequences of external causes” was the most common registration (35%) followed by “Factors influencing health status and contact with health services” (19%), “Symptoms, signs and abnormal clinical and laboratory findings, not elsewhere classified” (14%), and “Diseases of the circulatory system” (9%) (Table 2).

**Table 2:** The first diagnosis registered at hospital for patients hospitalized within 12 h following an emergency call, according to emergency call categorization as call_specific_ or call_unclear_ given as number (*N*) and percent (%)

Main chapter of ICD-10	Call_specific_	Call_unclear_	Total
	55,557 (83.2%)	11,233 (16.8%)	66,790
I. Certain infectious and parasitic diseases	586 (1.1)	466 (4.2)	1,052 (1.6)
II. Neoplasms	142 (0.3)	89 (0.8)	231 (0.4)
III. Diseases of the blood and blood forming organs and certain disorders involving the immune mechanism	207 (0.4)	89 (0.8)	296 (0.4)
IV. Endocrine, nutritional and metabolic diseases	927 (1.7)	552 (4.9)	1,479 (2.2)
V. Mental and behavioural disorders	1,738 (3.1)	454 (4.0)	2,192 (3.3)
VI. Diseases of the nervous system	1,470 (2.7)	287 (2.6)	1,757 (2.6)
VII. Diseases of the eye and adnexa	29 (0.1)	5 (<0.1)	34 (0.1)
VIII. Diseases of the ear and mastoid process	72 (0.1)	111 (1.0)	183 (0.3)
IX. Diseases of the circulatory system	5,049 (9.1)	1,006 (9.0)	6,055 (9.1)
X. Diseases of the respiratory system	3,259 (5.9)	511 (4.6)	3,770 (5.6)
XI. Diseases of the digestive system	1,786 (3.2)	372 (3.3)	2,158 (3.2)
XII. Diseases of the skin and subcutaneous tissue	97 (0.2)	38 (0.3)	135 (0.2)
XIII. Diseases of the musculoskeletal system and connective tissue	981 (1.8)	319 (2.8)	1,300 (2.0)
XIV. Diseases of the genitourinary system	886 (1.6)	323 (2.9)	1,209 (1.8)
XV. Pregnancy, childbirth and puerperium	332 (0.6)	22 (0.2)	354 (0.5)
XVI. Certain conditions originating in the perinatal period	3 (<0.1)	0 (0.0)	3 (<0.1)
XVII. Congenital malformations, deformations and chromosomal abnormalities	12 (<0.1)	1 (<0.1)	13 (<0.1)
XVIII. Symptoms, signs and abnormal clinical and laboratory findings, not elsewhere classified	7,987 (14.4)	3,052 (27.2)	11,039 (16.5)
XIX. Injury, poisoning and certain other consequences of external causes	19,237 (34.6)	1,348 (12.0)	20,585 (30.8)
XX. External causes of morbidity and mortality	55 (0.1)	4 (<0.1)	59 (0.1)
XXI. Factors influencing health status and contact with health services	10,702 (19.3)	2,184 (19.4)	12,886 (19.3)
XXII. Codes for special purposes	0 (0.0)	0 (0.0)	0 (0.0)

### Effect of emergency call categorization on mortality

**Fig. 3:**
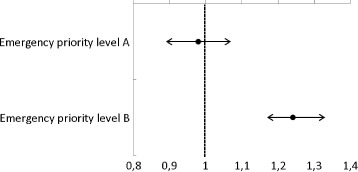
Incidence rate ratio (IRR) and 95% confidence interval (95% CI) for death according to the categorization of emergency calls as call_unclear_ compared to call_specific_ stratified by emergency level

Overall, there were 10,728 deaths in the study population, corresponding to an incidence rate of 160 deaths per 1,000 person years at risk (Table 3). The effect of emergency call categorization as call_unclear_ was modified by emergency priority level (*p*-value < 0.001 for the interaction between emergency priority level and categorization). When stratifying the analysis on emergency priority levels, there was no effect of emergency call categorization as call_unclear_ versus call_specific_ on mortality for individuals with emergency priority level A calls (Incidence Rate Ratio (IRR) = 0.93 (95% CI 0.85–1.01) for the semi adjusted model and IRR = 1.00 (95% CI 0.90–1.09) for the fully adjusted model). On the contrary, we found a positive association for individuals with emergency priority level B calls (IRR = 1.25 (95% CI 1.17–1.33) for the model adjusted for age and gender and IRR = 1.26 (95% CI 1.18–1.36) for the fully adjusted model) (Fig. 3).

**Table 3:** Descriptive analysis of mortality rates per 1,000 person years for individuals where emergency call was categorized as call_unclear_ vs call_specific_

Variable	Level	N_all_	N_dead_	PY	Rate	95% CI
Overall	-	97,293	10,728 (11%)	66,818	161	158; 164
Emergency priority level A^a^	Unclear problem	3,531	534 (15%)	2,367	226	207; 246
	Specific problem	40,852	6,087 (15%)	26,742	228	222; 233
Emergency priority level B^b^	Unclear problem	12,947	1,380 (11%)	9,602	144	136; 152
	Specific problem	39,963	2,727 (7%)	28,108	97	93; 101

Overall and stratified by emergency priority level. *N* number of individuals, *N*_*dead*_ number of deaths, *PY* person years, *rate* rate of deaths per 1,000 person years, *95% CI* 95% confidence interval for the rate of deaths per 1,000 person years. ^a^emergency priority level A is life threatening or potentially life threatening symptoms ^b^Emergency priority level B is acute but not life threatening symptoms (the medical dispatchers assessment of the patient)

## Discussion

We identified patients’ age, ethnicity, time of day, and day of week being significant predictors for emergency call categorization as call_unclear_. Patients brought to hospital within 12 h following an emergency call categorized as call_unclear_ had a high proportion of unspecific diagnoses registered at hospital, compared to patients with emergency call categorization as call_specific_. For emergency priority level A calls, categorization had no effect on mortality, whereas an association was observed for emergency priority level B calls, with an estimated IRR of 1.26 (95% CI 1.18–1.36) in the fully adjusted model.

### Predictors of unclear problems

Medical emergency calls are extremely critical communication situations where medical dispatchers’ interpretation of the presented problems takes place in a non-visual environment and without having the opportunity to monitor the patient directly. This is challenged by barriers such as the caller’s emotions and ability to explain the character of the problem [23]. Often the caller is not the patient him/herself adding to the complexity, as shown in a study of emergency calls where the caller was the patient in 7% of calls only [24]. Older age being associated with unclear problem categorization may be explained by a reduced capability of exchanging information quickly and precisely, but is yet to be explored. Nevertheless, older patients may be specifically vulnerable in terms of receiving correct pre-hospital management, like investigated by Hettinger et al. They found that subjects at 65 years or above were at increased risk for admission to hospital and death, in certain dispatch codes, including the code “Sick Person-Unknown Status/other codes not applicable [25].” Being non-Danish may complicate emergency calls due to language barriers, regardless of ethnicity [26]. This could affect the level of EMS response provided. However, one study investigating language disparities in patients transported by EMS found no association between being nonnative speaking and call priority or EMS transport time [27]. The daytime and weekdays being associated with emergency call categorization as call_unclear_, could be caused by increased workload in these timeslots. On the other hand, the causes for access differ during the day [8]. Besides patient-related predictors, other factors may influence categorization of emergency calls. Danish medical dispatchers are health care professionals with different experience and education. Also, the use of the dispatch tool may likely differ, depending on implementation and the working practices at the EMDC [28].

### Effect of emergency call categorization on mortality

The overall mortality rates for patients with call_specific_ and call_unclear_ was 11.6% and 10.9%, respectively. We found no association between categorization of calls and mortality within emergency priority level A, which is likely due to the dispatching of the most urgent ambulance response in 96.1% of cases with call_unclear_ and 95.2% of cases with call_specific_. It is well known that pre-hospital time delay is associated with mortality and disability [29, 30] and this is supported by our data. The identified difference in mortality for patients categorized as call_unclear_ compared to call_specific_ among emergency priority level B calls is crucial and supported by Andersen et al. who investigated possible preventable deaths for patients with emergency priority level B-E calls within 24 h prior to death. Of 18 included calls, 7 were categorized as unclear problem [31]. Another study investigating sensitivity of stroke recognition through emergency calls found that among the unrecognized strokes, 47% were categorized as unclear problem and of those, only 35% received the most acute ambulance response [32]. Furthermore, agonal breathing is well known as a barrier to identify cardiac arrest [33].

### Hospital diagnoses for patients brought to hospital

A relatively high proportion of calls resulted in provision of a red response and the proportion of hospital admissions was high. This finding may differ from other systems, where call cultures are different, as seen by the differences in emergency call incidences across countries [28]. Our results of diagnoses for patients brought to the hospital following emergency calls are supported by a recent study from another Danish region [34]. Importantly, our study demonstrated a high proportion of “unspecific” diagnoses registered at hospital for patients with call_unclear_ compared to patients with call_specific_. However, the proportions of patients who were registered with “Diseases of the circulatory system” and “Diseases of the nervous system” were as high for the call_unclear_ as for the call_specific_ patients. This finding confirms the underlying premise for pre-hospital emergency conditions, which seldom present themselves as “textbook examples.” Studies of the challenges in handling emergency calls by medical dispatchers would be beneficial.

### Implications

Altogether, our results shed light on the complexity of emergency call handling, but also implicate a need for further improvement. The identified predictors may help medical dispatchers in being alert when the callers are old, non-native or presenting with more comorbidity. Educational interventions at the dispatch centers may improve the call handling, but also the underlying supportive tools are modifiable. Follow up on the unclear cases in terms of true underlying conditions would provide valuable insights into which patients that are most susceptible for possible adverse consequences of not being recognized early, and this could implicate an addition or adjustment of questions in the dispatch tool.

We found a higher mortality rate for patients with emergency priority level B calls categorized as call_unclear_ compared to call_specific_. This result could imply lowering the threshold for dispatching a high level ambulance response when the call is considered unclear. Some degree of over-triage is expected and unavoidable [35]. On the other hand a “benefit of the doubt” approach could hinder the adequate response to other patients in need for an ambulance as there is an increasing demand and limited resources for ambulance services [36].

### Limitations

Our study has several limitations. The amount of missing CRS numbers for individuals for whom an emergency call was performed may result in a risk of selection bias. The analysis does not include factors related to the dispatcher. The professional background of the dispatcher, age, gender, and experience might affect emergency call categorization. However, in the study period, no data regarding the dispatchers were recorded at the EMDC. Furthermore, the data contain information about the patients, which are not always the caller. Data concerning the caller might affect our analysis of predictors. However, we do not have exact information about the type of caller. We compared the emergency calls categorized as “unclear problem” with emergency calls with symptom specific categories, which is a diverse group of symptoms/situations. However, our analyses were stratified into the two emergency priority levels to make them more comparable.

## Conclusion

Age, ethnicity, time of day, and day of week were significant predictors of emergency call categorization as “unclear problem”. “Unclear problem” categorization was not associated with mortality for emergency priority level A calls, but a higher mortality was observed for emergency priority level B calls. Decreasing the threshold for dispatching a high level EMS response in calls categorized as “unclear problem” assessed as emergency priority level B should be considered, but would increase over triage.

## Abbreviations

CI: Confidence interval
CRS: Civil registration system
EMDC: Emergency medical dispatch center
EMS: Emergency medical Services
ICD-10: International classification of diseases, version 10
IRR: Incidence rate ratio
OR: Odds ratio

## References

[1] Monsieurs KG, Nolan JP, Bossaert LL (2015). European resuscitation council guidelines for resuscitation 2015: section 1. Executive summary. Resuscitation.

[2] Berdowski J, Beekhuis F, Zwinderman AH, Tijssen JGP, Koster RW (2009). Importance of the first link: description and recognition of an out-of-hospital cardiac arrest in an emergency call. Circulation.

[3] Dami F, Heymann E, Pasquier M, Fuchs V, Carron P-N, Hugli O (2015). Time to identify cardiac arrest and provide dispatch-assisted cardio-pulmonary resuscitation in a criteria-based dispatch system. Resuscitation.

[4] Lewis M, Stubbs BA, Eisenberg MS (2013). Dispatcher-assisted cardiopulmonary resuscitation: time to identify cardiac arrest and deliver chest compression instructions. Circulation.

[5] Herlitz J, Wireklintsundström B, Bång A, Berglund A, Svensson L, Blomstrand C (2010). Early identification and delay to treatment in myocardial infarction and stroke: differences and similarities. Scand J Trauma Resusc Emerg Med.

[6] Vaillancourt C, Charette M, Kasaboski A (2015). Cardiac arrest diagnostic accuracy of 9-1-1 dispatchers: a prospective multi-center study. Resuscitation.

[7] Andersen MS, Johnsen SP, Sørensen JN, Jepsen SB, Hansen JB, Christensen EF (2013). Implementing a nationwide criteria-based emergency medical dispatch system: a register-based follow-up study. Scand J Trauma Resusc Emerg Med.

[8] Møller TP, Ersbøll AK, Tolstrup JS (2015). Why and when citizens call for emergency help: an observational study of 211,193 medical emergency calls. Scand J Trauma Resusc Emerg Med.

[9] Ellensen EN, Hunskaar S, Wisborg T, Zakariassen E (2014). Variations in contact patterns and dispatch guideline adherence between Norwegian emergency medical communication centres-a cross-sectional study. Scand J Trauma Resusc Emerg Med.

[10] Grusd E, Kramer-Johansen J (2016). Does the Norwegian emergency medical dispatch classification as non-urgent predict no need for pre-hospital medical treatment? An observational study. Scand J Trauma Resusc Emerg Med.

[11] Culley LL, Henwood DK, Clark JJ, Eisenberg MS, Horton C (1994). Increasing the efficiency of emergency medical services by using criteria based dispatch. Ann Emerg Med.

[12] Schagerlind L, Örtenwall P, Widgren BR, Taube M, Asplén B, Örninge P, Khorram AM (2013). Validation of Swedish emergency medical index in trauma patients. JEDM.

[13] Pedersen CB (2011). The Danish civil registration system. Scand J Public Health.

[14] Jensen VM, Rasmussen AW (2011). Danish education registers. Scand J Public Health.

[15] Baadsgaard M, Quitzau J (2011). Danish registers on personal income and transfer payments. Scand J Public Health.

[16] Petersson F, Baadsgaard M, Thygesen LC (2011). Danish registers on personal labour market affiliation. Scand J Public Health.

[17] Schmidt M, Schmidt SAJ, Sandegaard JL, Ehrenstein V, Pedersen L, Sørensen HT (2015). The Danish national patient registry: a review of content, data quality, and research potential. Clin Epidemiol.

[18] Helweg-Larsen K (2011). The Danish register of causes of death. Scand J Public Health.

[19] Wallach-Kildemoes H, Andersen M, Diderichsen F, Lange T (2013). Adherence to preventive statin therapy according to socioeconomic position. Eur J Clin Pharmacol.

[20] Charlson ME, Pompei P, Ales KL, MacKenzie CR (1987). A new method of classifying prognostic comorbidity in longitudinal studies: development and validation. J Chronic Dis.

[21] American Medical Association (2015). 2016 ICD-10-CM: the complete official codebook.

[22] Ulm K (1990). A simple method to calculate the confidence interval of a standardized mortality ratio (SMR). Am J Epidemiol.

[23] Alfsen D, Møller TP, Egerod I, Lippert FK (2015). Barriers to recognition of out-of-hospital cardiac arrest during emergency medical calls: a qualitative inductive thematic analysis. Scand J Trauma Resusc Emerg Med.

[24] Karlsten R, Elowsson P (2004). Who calls for the ambulance: implications for decision support. A descriptive study from a Swedish dispatch centre. Eur J Emerg Med.

[25] Hettinger AZ, Cushman JT, Shah MN, Noyes K (2013). Emergency medical dispatch codes association with emergency department outcomes. Prehospital Emerg Care.

[26] Meischke HW, Calhoun RE, Yip M-P, Tu S-P, Painter IS (2013). The effect of language barriers on dispatching EMS response. Prehospital Emerg Care.

[27] Weiss NR, Weiss SJ, Tate R, Oglesbee S, Ernst AA (2015). Language disparities in patients transported by emergency medical services. Am J Emerg Med.

[28] Møller TP, Andréll C, Viereck S, Todorova L, Friberg H, Lippert FK (2016). Recognition of out-of-hospital cardiac arrest by medical dispatchers in emergency medical dispatch centres in two countries. Resuscitation.

[29] Terkelsen CJ, Sørensen JT, Maeng M (2010). System delay and mortality among patients with STEMI treated with primary percutaneous coronary intervention. JAMA.

[30] Fonarow GC, Smith EE, Saver JL (2011). Improving door-to-needle times in acute ischemic stroke: the design and rationale for the american heart association/american stroke Association’s target: stroke initiative. Stroke J Cereb Circ.

[31] Andersen MS, Johnsen SP, Hansen AE (2014). Preventable deaths following emergency medical dispatch - an audit study. Scand J Trauma Resusc Emerg Med.

[32] Viereck S, Møller TP, Iversen HK, Christensen H, Lippert F (2016). Medical dispatchers recognise substantial amount of acute stroke during emergency calls. Scand J Trauma Resusc Emerg Med.

[33] Fukushima H, Imanishi M, Iwami T (2015). Implementation of a dispatch-instruction protocol for cardiopulmonary resuscitation according to various abnormal breathing patterns: a population-based study. Scand J Trauma Resusc Emerg Med.

[34] Christensen EF, Larsen TM, Jensen FB, et al. Diagnosis and mortality in prehospital emergency patients transported to hospital: a population-based and registry-based cohort study. BMJ Open. 2016;6:e011558. 10.1136/bmjopen-2016-011558 PMC4947831 27377636

[35] Cameron PA, Gabbe BJ, Smith K, Mitra B (2014). Triaging the right patient to the right place in the shortest time. Br J Anaesth.

[36] Pittet V, Burnand B, Yersin B, Carron P-N (2014). Trends of pre-hospital emergency medical services activity over 10 years: a population-based registry analysis. BMC Health Serv Res.

